# A case of autoimmune pulmonary alveolar proteinosis during the course of treatment of rapidly progressive interstitial pneumonia associated with anti-MDA5 antibody-positive dermatomyositis

**DOI:** 10.1186/s12890-024-02989-9

**Published:** 2024-04-08

**Authors:** Masakiyo Yatomi, Keiichi Akasaka, Shintaro Sato, Mizuki Chida, Mio Kanbe, Hiru Sawada, Itaru Yokota, Ikuo Wakamatsu, Sohei Muto, Mari Sato, Kochi Yamaguchi, Yosuke Miura, Hiroaki Tsurumaki, Reiko Sakurai, Kenichiro Hara, Yasuhiko Koga, Noriaki Sunaga, Hideaki Yamakawa, Hidekazu Matsushima, Sahori Yamazaki, Yukie Endo, Sei-ichiro Motegi, Takeshi Hisada, Toshitaka Maeno

**Affiliations:** 1 Division of Allergy and Respiratory Medicine, Integrative Center of Internal Medicine, Gunma University Hospital, 3-39-15 Showa-Machi, Maebashi, Gunma 371- 8511 Japan; 2 Department of Respiratory Medicine, Saitama Red Cross Hospital, 1-5, Shintoshin, Chuo-Ku, Saitama, 330-8553 Japan; 3 Department of Dermatology, Gunma University Graduate School of Medicine, Gunma University Hospital, 3-39-15 Showa-Machi, Maebashi, Gunma 371- 8511 Japan; 4 Oncology Center, Gunma University Hospital, 3-39-15 Showa-Machi, Maebashi, Gunma 371-, 8511 Japan; 5 Gunma University Graduate School of Health Sciences, 3-39-22 Showa-Machi, Maebashi, Gunma 371-8514 Japan

**Keywords:** Melanoma differentiation-associated gene 5, Autoimmune pulmonary alveolar proteinosis, Granulocyte–macrophage colony-stimulating factor, Granulocyte–macrophage colony-stimulating factor antibodies, Rapidly progressive interstitial lung disease

## Abstract

**Background:**

Autoimmune pulmonary alveolar proteinosis (APAP) is a diffuse lung disease that causes abnormal accumulation of lipoproteins in the alveoli; however, its pathogenesis remains unclear. Recently, APAP cases have been reported during the course of dermatomyositis. The combination of these two diseases may be coincidental; however, it may have been overlooked because differentiating APAP from a flare-up of interstitial pneumonia associated with dermatomyositis is challenging. This didactic case demonstrates the need for early APAP scrutiny.

**Case presentation:**

A 50-year-old woman was diagnosed with anti-melanoma differentiation-associated gene 5 (anti-MDA5) antibody-positive dermatitis and interstitial pneumonia in April 2021. The patient was treated with corticosteroids, tacrolimus, and cyclophosphamide pulse therapy for interstitial pneumonia complicated by MDA5 antibody-positive dermatitis, which improved the symptoms and interstitial pneumonia. Eight months after the start of treatment, a new interstitial shadow appeared that worsened. Therefore, three additional courses of cyclophosphamide pulse therapy were administered; however, the respiratory symptoms and interstitial shadows did not improve. Respiratory failure progressed, and 14 months after treatment initiation, bronchoscopy revealed turbid alveolar lavage fluid, numerous foamy macrophages, and numerous periodic acid–Schiff-positive unstructured materials. Blood test results revealed high anti-granulocyte–macrophage colony-stimulating factor (GM-CSF) antibody levels, leading to a diagnosis of APAP. The patient underwent whole-lung lavage, and the respiratory disturbance promptly improved. Anti-GM-CSF antibodies were measured from the cryopreserved serum samples collected at the time of diagnosis of anti-MDA5 antibody-positive dermatitis, and 10 months later, both values were significantly higher than normal.

**Conclusions:**

This is the first report of anti-MDA5 antibody-positive dermatomyositis complicated by interstitial pneumonia with APAP, which may develop during immunosuppressive therapy and be misdiagnosed as a re-exacerbation of interstitial pneumonia. In anti-MDA5 antibody-positive dermatomyositis, APAP comorbidity may have been overlooked, and early evaluation with bronchoalveolar lavage fluid and anti-GM-CSF antibody measurements should be considered, keeping the development of APAP in mind.

## Background

Granulocyte–macrophage colony stimulating factor (GM-CSF), which is a major regulator of granulocyte and macrophage lineage populations, has been implicated in inflammatory, infectious, and autoimmune diseases [1]. Pulmonary alveolar proteinosis (PAP) is a lung disease that results in impaired clearance of surfactants and the accumulation of surfactant-derived substances in the alveoli. Autoimmune PAP (APAP), which accounts for 89% of PAP cases, occurs when anti-GM-CSF antibodies (GMAb) reduce the phagocytic ability of alveolar macrophages, resulting in an inability to process old surfactants in the alveoli and induce surfactant accumulation. The characteristic findings of PAP on computed tomography (CT) include a thickened interlobular septal wall commonly referred to as a crazy-paving appearance, geographic distribution, and subpleural sparing [2, 3].

In addition to the CT imaging findings, GMAb measurement is necessary for the diagnosis of APAP [4, 5]. The recently developed enzyme-linked immunosorbent assay test kit for GMAb ensures a simple testing procedure with high sensitivity and specificity, which is reliable for the diagnosis of APAP and the differential diagnosis of other lung diseases [6]. Commercial measurements are currently available in Japan. GMAb is rarely elevated in non-APAP diseases and has been reported to be elevated in diseases, such as myasthenia gravis, sarcoidosis, various interstitial pneumonias, and dust pneumonia [7–9].

In general, APAP is rarely associated with autoimmune diseases, such as dermatomyositis [10], and several reports have suggested GMAb, anti-melanoma differentiation-associated protein-5 (MDA5), and anti-aminoacyl-tRNA synthetase (ARS) antibodies to be grossly exclusive [6].

However, in the present study, we encountered a case of rapidly progressive interstitial lung disease (RP-ILD) complicated by anti-MDA5 antibody-positive dermatomyositis, which developed into respiratory failure due to the re-aggravation of diffuse ground-glass opacity (GGO) during treatment, leading to the diagnosis of APAP. We examined the mechanism of APAP development based on the clinical course and GMAb trends. In addition, we analyzed previously reported cases of APAP complicated by the presence of dermatomyositis-related antibodies, including the present case, and discussed new causes of APAP.

### Case presentation

**Fig. 1:**
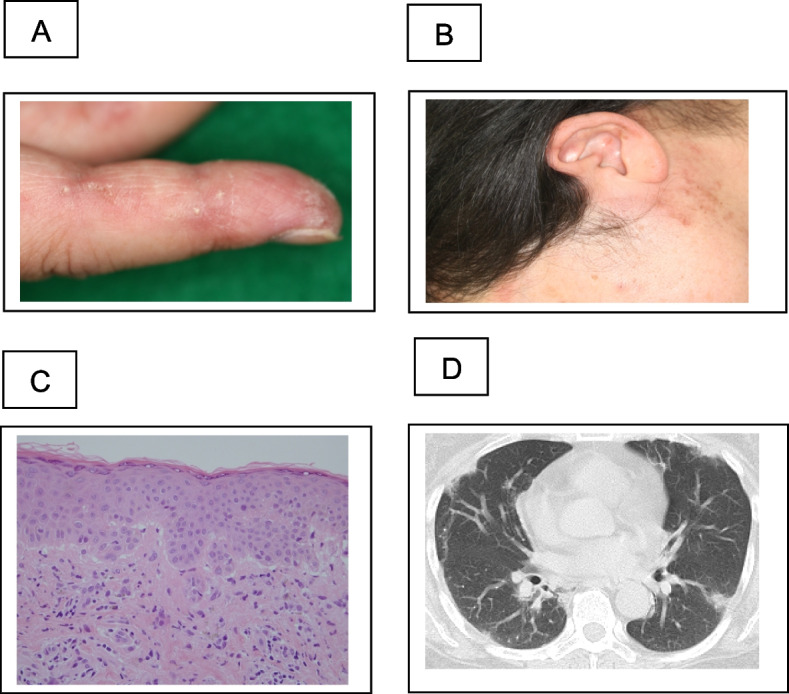
Characteristic findings of anti-MDA5 antibody-positive dermatomyositis. **A** Reverse Gottron’s sign and **B** exudative erythema, representing characteristic findings of anti-melanoma differentiation-associated gene 5 antibody-positive dermatomyositis. **C** Dermatopathological findings showed liquid degeneration of the epidermal basement membrane, dermal mucin deposition, and perivascular inflammatory cell infiltration of the dermis. **D** Computed tomography (CT) findings on first admission. Infiltrative and interstitial shadows are observed just below the pleura

**Fig. 2:**
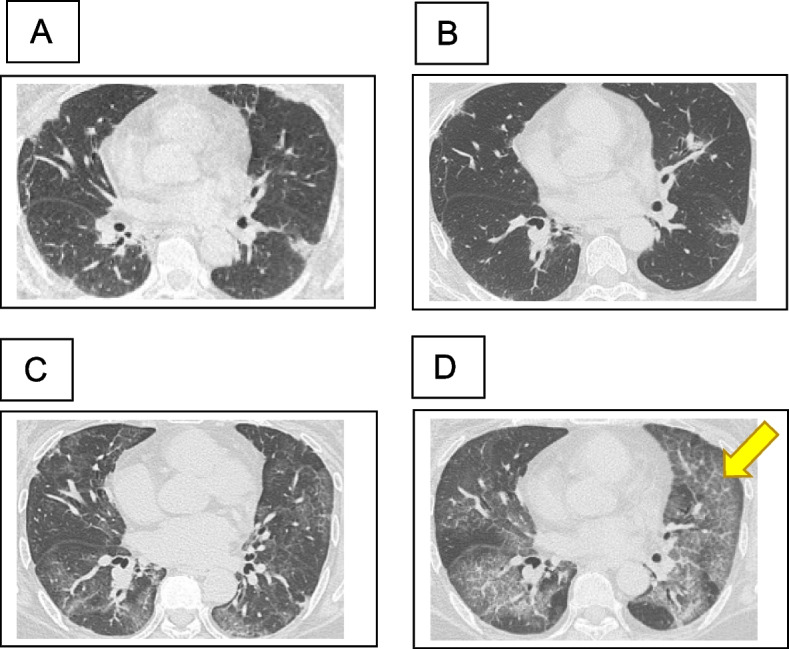
Course of findings of interstitial pneumonia on chest computed tomography (CT). **A** At the diagnosis of dermatomyositis, **B** 2 months after initiation of treatment, **C** 8 months after initiation of treatment, and **D** 14 months after initiation of treatment (before bronchoscopy). On initial admission (**A**), interstitial shadows were observed just below the pleura in both lungs. At discharge 2 months later, the interstitial shadows temporarily improved (**B**). However, 8 months later (**C**), new ground-glass shadows appeared in both lungs. Fourteen months later (**D**), the ground-glass shadows were further aggravated, as indicated by the arrow

**Fig. 3:**
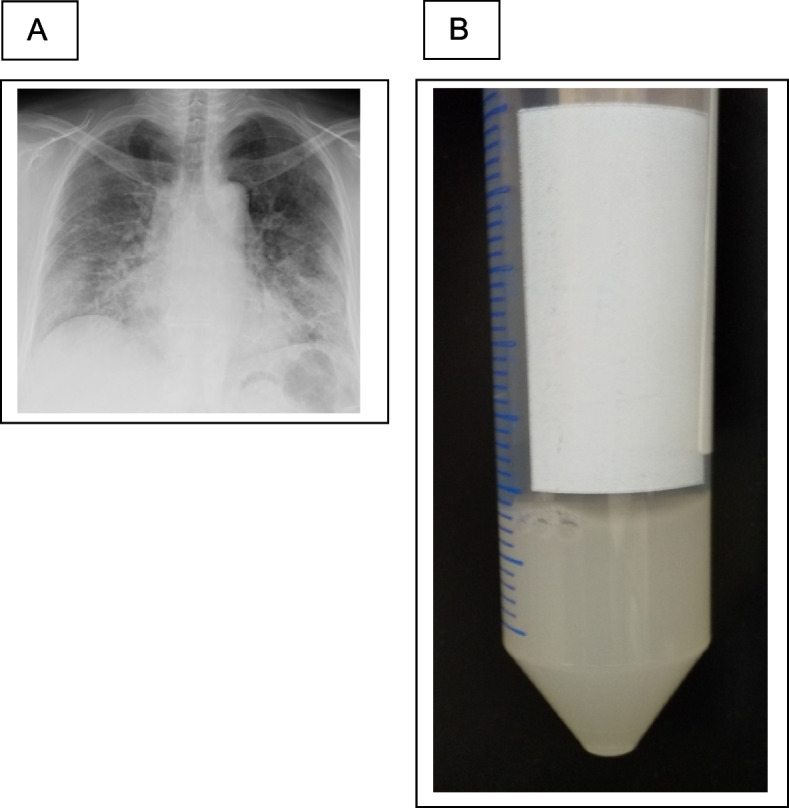
Chest x-ray on admission for bronchoscopy and bronchoalveolar lavage fluid (BALF). **A** Chest radiograph before bronchoscopy showing ground-glass shadows in both lungs. **B** The collected alveolar lavage fluid was thick and milky white in color

**Fig. 4:**
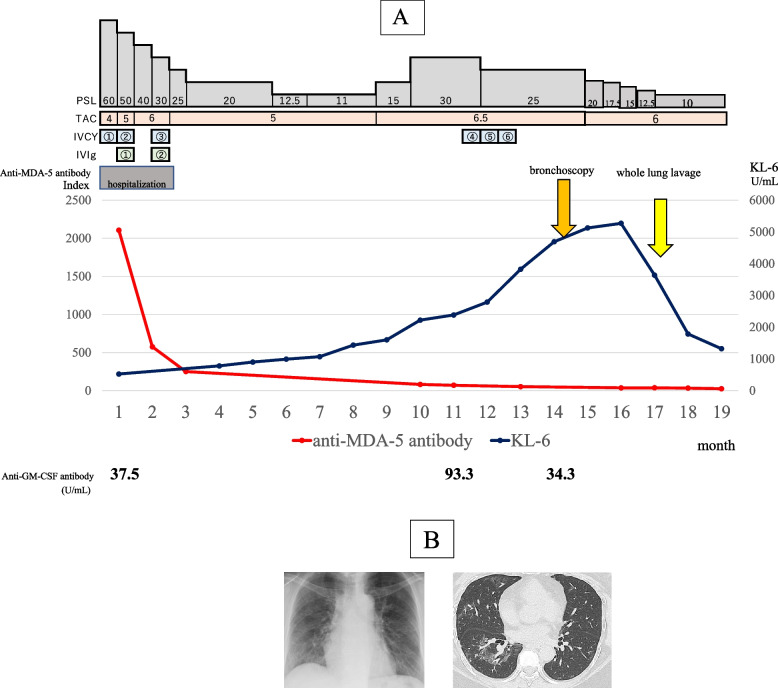
Clinical course and images after whole lung lavage. **A** Changes in therapeutic agents and laboratory values. Anti-MDA-5 antibody levels decreased after treatment with anti-melanoma differentiation-associated gene 5 antibody-positive dermatomyositis complicated by interstitial pneumonia, but KL-6 tended to increase after 8 months. After diagnosis of APAP, prednisolone (PSL) was tapered off and whole-lung lavage was performed, after which KL-6 decreased. The anti-GM-CSF antibody titer was high from the first admission, suggesting that it remained high until the onset of APAP. **B** Chest radiograph and computed tomography (CT) after whole lung lavage. Significant improvement in ground-glass shadows in both lungs was found after whole lung lavage. PSL, prednisolone; TAC, tacrolimus; IVCY, intravenous cyclophosphamide; IVIg, intravenous immunoglobulin; MDA-5, melanoma differentiation-associated gene 5; GM-CSF, granulocyte–macrophage colony-stimulating factor; KL-6, Krebs von den Lungen 6; APAP, autoimmune pulmonary alveolar proteinosis

A woman in her 50 s developed a skin rash and dry cough in November 2020. In April 2021, the patient was diagnosed with anti-MDA5 antibody-positive dermatomyositis at the Department of Dermatology at our hospital. The physical and skin findings at the time of diagnosis are shown in Fig. 1A, B, and C. Histopathology of the skin biopsy was consistent with dermatomyositis. The serum anti-MDA5 antibody level was 2,105 U/mL, and ferritin markedly elevated at 1,527 ng/mL. Additionally, infiltrative and interstitial shadows were observed at the pleural margins of both lungs on CT (Fig. 1D). The polymerase chain reaction test result for severe acute respiratory syndrome coronavirus type 2 (SARS-CoV-2) in saliva was negative. Although respiratory failure was not observed, RP-ILD was suspected, and the patient was immediately admitted to the hospital, where high-dose prednisolone therapy (1 mg/kg), tacrolimus administration (4 mg/day), and cyclophosphamide pulse therapy (500 mg/m^2^) were initiated. After treatment with high-dose intravenous immunoglobulin (IVIG: 1 g/day for 5 days), the dermatomyositis and interstitial shadows improved. The patient was discharged from the hospital 2 months after admission because her condition improved. The prednisolone dose was reduced to 25 mg/day, and cyclophosphamide pulse therapy was terminated after three sessions owing to pronounced leukopenia and thrombocytopenia following cyclophosphamide administration. Figure 2 shows the changes in the CT images. However, 8 months after admission, the GGO worsened with Krebs von den Lungen (KL-6) elevation, and respiratory failure developed. Although the anti-MDA5 antibody levels tended to decrease (Fig. 4A) and there was no worsening of myositis, we judged that this was another exacerbation of interstitial pneumonia complicated by anti-MDA5 antibody-positive dermatomyositis. Hence, we treated the patient with an increased dose of prednisolone (30 mg/day) and tacrolimus (6.5 mg/day). Furthermore, we repeated cyclophosphamide pulse therapy as an outpatient for three courses. Despite these treatments, the GGO worsened, and respiratory failure developed, further warranting home oxygen therapy. Fourteen months after the onset of dermatomyositis, the patient was hospitalized for further examination. Upon admission, chest radiography revealed frosted shadows in both lungs (Fig. 3A). CT showed a new diffuse crazy-paving appearance (Fig. 2D). Blood test results on admission showed elevated levels of KL-6, surfactant protein-D, and cytokeratin 19 fragment, while the ferritin level was low, and anti-MDA-5 antibodies tended to decrease (Table 1). Bronchoalveolar lavage was performed on the fourth day after admission, and 88 ml of 150 mL bronchalveolar lavage fluid (BALF), which was milky white, was collected (Fig. 3B). Further analysis of the cellular composition revealed macrophage predominance, with numerous foamy macrophages and a large amount of acidophilic unstructured material in the background (Table 2). Based on these examination results, a diagnosis of PAP was made. Subsequently, we measured GMAb, which was high at 34.3 U/mL, and finally diagnosed the patient with APAP. Thereafter, the prednisolone dose was reduced to 10 mg/day every two weeks to reduce the risk of post-procedural infection, and whole-lung lavage was performed 16 months after the onset of dermatomyositis. The clinical course of the patient is shown in Fig. 4A. After whole lung lavage, the GGO area on the CT scan decreased compared with that prior to whole lung lavage, shortness of breath improved, and the amount of oxygen needed for inhalation was markedly reduced (Fig. 4B). We later measured the GMAb in two serum samples stored in our hospital collected at the onset and 11 months after the onset of dermatomyositis and found the levels were both high at 37.5 U/mL and 93.3 U/mL, respectively (Fig. 4 A).

**Table 1:** Blood tests on admission for bronchoscopy

Hematology			Biochemistry		
White blood cells	**8,300**	/μL	Total protein	**6.7**	g/dL
Neutrophils	**91.4**	%	Serum albumin	**4**	g/dL
Eosinophils	**0.1**	%	Total bilirubin	**0.6**	mg/dL
Basophils	**0.2**	%	Aspartate transaminase	**20**	U/L
Monocytes	**1.1**	%	Alanine transaminase	**18**	U/L
Lymphocytes	**7.2**	%	Lactate dehydrogenase	**325**	U/L
Neutrophils	**7,586**	/μL	Alkaline phosphatase	**44**	U/L
Eosinophils	**8.3**	/μL	Creatine kinase	**40**	U/L
Basophils	**16.6**	/μL	Blood urea nitrogen	**15**	mg/dL
Monocytes	**91.3**	/μL	Creatinine	**0.71**	mg/dL
Lymphocytes	**597.6**	/μL	C-reactive protein	**0.07**	mg/dL
Hemoglobin	**16.8**	g/dL	Serum sodium	**145**	mEq/L
Platelets	**237,000**	/μL	Serum potassium	**4.6**	mEq/L
**Coagulation**			Serum chloride	**107**	mEq/L
Prothrombin time	**112**	%	Glucose	**174**	mg/dL
Activated partial thromboplastin time	**25.8**	second	Ferritin	**20.3**	ng/mL
D-dimer	**0.5**	µg/mL			
**Serology**					
Sialylated carbohydrate antigen KL-6	**4,689**	ng/mL			
Surfactant protein D	**211**	ng/mL			
Perinuclear anti-neutrophil cytoplasmic antibody	**< 1.0**	U/mL			
Classic anti-neutrophil cytoplasmic autoantibody	**< 1.0**	U/mL			
Cytokeratin 19 fragment	**12.7**	ng/mL			
Cytomegalovirus antigenaemia test	**(-)**				
Anti-Trichosporon asahii antibody	**(-)**				
anti-nuclear antibody	**< 40**				
β-Dglcan	**< 2.6**	pg/mL			

Blood tests on admission revealed marked elevation in KL-6 and SP-D levels, whereas ferritin and CRP levels were low

**Table 2:** Properties of bronchoalveolar lavage fluid and results of each test

Bronchoalveolar lavage fluid		
Recovery rate	58.7 (88/150 mL)	%
Total cells	45,000	/mL
Macrophages	76	%
Lymphocytes	12	%
Neutrophils	12	%
Eosinophils	0	%
Basophils	0	%
CD4/CD8	0.9	
General bacterial culture	Negative	
Antimicrobial culture	Negative	
Candida quantification	< 4 × 10^1^	copy/mL
Aspergillus quantification	< 4 × 10^1^	copy/mL
Pneumocystis determination	< 4 × 10^1^	copy/mL
Cytodiagnosis	no malignancy	

In the bronchoalveolar lavage fluid (BALF), antigen quantification testing of this alveolar lavage fluid for infectious agents showed that *Pneumocystis*, *Aspergillus*, and *Candida* were extremely few. Cellular fractionation showed an increased macrophage ratio, with no infectious or malignant findings

## Discussion

Since interstitial pneumonia complicated by anti-MDA5 antibody-positive dermatomyositis is often severe and results in respiratory failure due to RP-ILD, early implementation of potent immunosuppressive therapy should be considered. Cases not treated with prednisolone, cyclophosphamide, or tacrolimus have been reported to have a poor prognosis [11]. However, if appropriate treatment is administered in the acute phase and remission is achieved, the risk of relapse is lower, with a reported frequency of 33% [11].

During the treatment of anti-MDA5 antibody-positive interstitial pneumonia, we initially diagnosed the patient with GGO due to exacerbation and administered prednisolone and immunosuppressive drugs.

Anti-MDA5 antibody levels are generally considered indicators of dermatomyositis activity [12]. However, as shown in Fig. 4A, the anti-MDA5 antibody level steadily decreased, while KL-6 levels were elevated and diffuse GGO worsened. This discrepancy suggests the possibility of another diffuse lung disease rather than interstitial pneumonia complicated by anti-MDA5 antibody-positive dermatomyositis. Furthermore, since the patient’s condition did not improve and the GGO worsened despite increasing doses of prednisolone, additional treatment with three courses of cyclophosphamide pulse therapy, increasing doses of tacrolimus, and BALF in bronchoscopy was performed, which resulted in the diagnosis of APAP.

To our knowledge, there have been no previous reports of APAP in patients with dermatomyositis complicated by interstitial pneumonia who tested positive for anti-MDA5 antibodies. However, recently, several cases of APAP in interstitial pneumonia complicated by polymyositis and anti-ARS antibody-positive interstitial pneumonia have been reported [13, 14]. In this case, two serum samples from the patient were stored during the period between the onset of anti-MDA5 antibody-positive interstitial pneumonia and the diagnosis of APAP, which allowed us to measure GMAb in the sera and analyze the course of APAP development. Interestingly, as shown in Fig. 4, the GMAb levels were elevated at the onset of anti-MDA5 antibody-positive dermatomyositis complicated by interstitial pneumonia. A recent retrospective cohort study reported that corticosteroid administration may worsen the severity of APAP [15]. Corticosteroids promote surfactant synthesis from alveolar type II epithelial cells and may be administered in cases of early childbirth. Corticosteroids also decrease macrophage function [16, 17]. Thus, corticosteroid administration decreases the clearance capacity of the surfactant. In addition, tacrolimus selectively promotes the degradation of GM-CSF and messenger RNA, as well as decreases GM-CSF transcriptional activation [18, 19]. However, because the impact of tacrolimus' GM-CSF activity-lowering effect on APAP is not well understood, we continued to administer tacrolimus, which is an important therapeutic agent for the management of anti-MDA-5 antibody-positive dermatomyositis.

This is a case of anti-MDA5 antibody-positive dermatomyositis with bystander GMAb without APAP development at the initial diagnosis, which appeared to manifest and develop during immunosuppressive therapy with prednisolone and tacrolimus as intensified therapy. The antibody concentration did not appear to be suppressed by immunosuppressive therapy but rather increased to 93.3 U/mL and remained at a high value, consistent with disease activity. Although GMAb levels do not necessarily reflect APAP activity [9, 20], in the present case, the GMAb levels increased despite heavy immunosuppressive therapy, indicating that high APAP activity was maintained. Although corticosteroids were tapered off, they were administered for a long period of time, and since the GMab levels remained elevated, long-term corticosteroid administration may have increased the alveolar surfactant levels and exacerbated the alveolar macrophage dysfunction, thus increasing the risk of developing APAP.

GM-CSF may be involved in the activity of dermatomyositis [21]; moreover, GM-CSF inhalation therapy has been shown to improve alveolar–arterial O_2_ levels in patients with APAP [22]. Although the mechanism of GMAb formation is not yet known [7], it is interesting to note that the patient developed APAP even under immunocompromised conditions owing to the administration of immunosuppressive drugs. GM-CSF is an inflammatory cytokine involved in the pathogenesis of autoimmune diseases. The pathophysiology is easy to understand if we consider that excessive production of GM-CSF occurs during periods of high activity of dermatomyositis, and excessive production of GMAb occurs as a self-defense mechanism against excessive GM-CSF production. However, in the present case, GMAb levels increased even when dermatomyositis activity decreased. Furthermore, at that time, since immunosuppressive therapy had already been administered, total IgG levels were expected to decrease, and GMAb was expected to decrease accordingly; however, the measured GMAb remained high.

Nakata et al. measured GMAb in 162 cases of miscellaneous lung disease, except for APAP, over 7 years and 3 months from January 2012 to May 2018 in Japan and found that five cases had cut-off levels (> 1.65 U/mL-1) or higher, but all cases were negative for anti-MDA-5 or anti-ARS antibody, which is grossly exclusive [6]. However, one MDA-5 positive case (this report) and three ARS-positive cases were reported in the 7-year period from 2018 to 2024, all from Japan [10, 12, 13]. The pathophysiology, which is grossly exclusive, is unknown. To our knowledge, no papers, including articles in basic medicine, have discussed the total exclusivity of ARS, MDA-5, and GM-CSF for various antibodies. In fact, it is not inconsistent to have patients positive for the three antibodies; rather, they may have been missed by not measuring the GMAb. Additionally, these patients were likely to have developed APAP during immunosuppressive therapy.

In 2020, Sato et al. reported seven cases of APAP complicated by connective tissue diseases [13]; however, this is the first case of APAP complicated by dermatomyositis positive for anti-MDA5 antibody. Furthermore, a report highlighted secondary PAP (SPAP) in patients with anti-MDA5 antibody-positive dermatomyositis who developed SPAP while receiving immunosuppressive agents [10]. SPAP is defined as PAP occurring alongside an underlying condition that tests negative for GMAb [23]. The etiology of SPAP typically involves macrophage dysfunction linked to the underlying disease, often hematologic in nature, such as myelodysplastic syndrome. Although autoimmune diseases may also be implicated, they are less frequently observed. Administration of immunosuppressive agents, such as corticosteroids, common in autoimmune diseases, may exacerbate macrophage dysfunction, potentially leading to the development of SPAP even without elevated GMAb levels. However, this case was diagnosed as APAP due to high GMAb levels associated with anti-MDA5 antibody-positive dermatomyositis. Nonetheless, it shares similarities with SPAP in that it emerged during the administration of immunosuppressive therapy.

The most frequent cause of worsening GGO during myositis-associated interstitial pneumonia id concomitant infection, including pneumocystis carinii pneumonia, cytomegalovirus, or coronavirus disease 2019. The second most common cause is drug-induced pneumonia, such as methotrexate or biological agents, and the third is aggravation of interstitial pneumonia due to the underlying disease. Although much less common, PAP should be advocated as the fourth leading cause of immunosuppressive therapy. Importantly, further accumulation of such cases and their analyses are required in the future. Furthermore, since it may be difficult to differentiate PAP from the exacerbation of underlying interstitial pneumonia, we propose the importance of early bronchoscopic evaluation, such as BALF and GMAb measurement, for the diagnosis of APAP.

## Conclusions

This is the first report of APAP in a patient with anti-MDA5 antibody-positive dermatomyositis complicated by interstitial pneumonia. Thus, early measurement of BALF and anti-GM-CSF antibodies is important for the diagnosis of APAP. APAP may manifest during immunosuppressive treatment.

## Data Availability

The datasets used and/or analyzed during the current study available from the corresponding author on reasonable request.  All data generated or analyzed during this study are included in this published article and its supplementary information files.

## Abbreviations

MDA-5: Melanoma differentiation-associated gene 5
APAP: Autoimmune pulmonary alveolar proteinosis
GF-CSF: Granulocyte–macrophage colony-stimulating factor
GMAb: Anti-GM-CSF antibodies
RP-ILD: Rapidly progressive interstitial lung disease
BALF: Bronchoalveolar lavage fluid
GGO: Ground-glass opacity
